# *Peptoniphilus genitalis* sp. nov. and *Mobiluncus massiliensis* sp. nov.: Novel Bacteria Isolated from the Vaginal Microbiome

**DOI:** 10.1007/s00284-023-03584-7

**Published:** 2024-02-19

**Authors:** Linda Abou Chacra, Marion Bonnet, Mégane Heredia, Gabriel Haddad, Nicholas Armstrong, Stéphane Alibar, Florence Bretelle, Florence Fenollar

**Affiliations:** 1 Aix-Marseille Université, IRD, AP-HM, SSA, VITROME, Marseille, France; 2 IHU-Méditerranée Infection, Marseille, France; 3 Aix-Marseille Université, IRD, AP-HM, MEPHI, Marseille, France; 4 Department of Gynaecology and Obstetrics, Gynépole, La Conception, AP-HM, Marseille, France

## Abstract

The strains Marseille-Q7072^T^ (= CSUR Q7072^T^ = CECT 30604^ T^) and Marseille-Q7826^T^ (= CSUR Q7826^T^ = CECT 30727^ T^) were isolated from vaginal samples. As MALDI-TOF mass spectrometry failed to identify them, their genomes were directly sequenced to determine their taxogenomic identities. Both strains are anaerobic without any oxidase and catalase activity. C_16:0_ is the most abundant fatty acid for both strains. Strain Marseille-Q7072^T^ is non-spore-forming, non-motile, Gram-stain-positive, and coccus-shaped, while strain Marseille-Q7826^T^ is non-spore-forming, motile, Gram-stain-variable, and curved rod-shaped. The genomic comparison of the Marseille-Q7072^T^ and Marseille-Q7826^T^ strains showed that all digital DNA–DNA hybridisation (dDDH) and mean orthologous nucleotide identity (OrthoANI) values were below published species thresholds (70% and 95–96%, respectively) with other closely related species with standing in nomenclature. Thus, we conclude that both strains are new bacterial species. Strain Marseille-Q7072^T^ is a new member of the *Bacillota* phylum, for which the name *Peptoniphilus genitalis* sp. nov. is proposed, while the Marseille-Q7826^T^ strain is a new member of the *Actinomycetota* phylum, for which the name *Mobiluncus massiliensis* sp. nov. is proposed.

**Supplementary Information:**

The online version contains supplementary material available at 10.1007/s00284-023-03584-7.

## Introduction

The vaginal microbiota is a complex ecosystem of microorganisms that inhabit the vagina and play a crucial role in women’s genital health [1]. This microbiota is dominated by lactobacilli, which are responsible for the production of lactic acid, thus maintaining an acidic vaginal pH that prevents colonisation by potentially harmful pathogens [1]. An imbalance in the composition of the vaginal microbiota, known as bacterial vaginosis, is associated with an increased risk of sexually transmitted infections, obstetric complications, and pelvic inflammatory diseases [2].

Despite the importance of the vaginal microbiota for women’s health, our understanding of its diversity and composition remains limited due to the difficulties in culturing and characterising the bacteria that make it up [3]. Next-generation sequencing methods have led to significant advances in the study of the vaginal microbiota [4], but these approaches do not always allow for a complete characterisation of the microorganisms present.

In this context, culturomics-based approaches have emerged as a powerful complementary method for analysing the vaginal microbiota, allowing for a more in-depth characterisation of the cultivable microorganisms that compose it [5]. As part of a complete characterisation of the vaginal microbiota by culturomics in women who gave birth prematurely and those who had repeated miscarriages, we isolated two new members of the genus *Peptoniphilus* and *Mobiluncus* from vaginal samples that did not match other species of their genus. These strains were designated Marseille-Q7072^T^ and Marseille-Q7826^T^, respectively.

In accordance with the polyphasic taxonogenomic strategy combining annotated whole genome, proteomic information extracted from MALDI-TOF mass spectrometry spectra, and phenotypic aspects, we present here the description of these two strains.

## Materials and Methods

### Ethical Considerations and Sampling

Two vaginal specimens sampled during patient care and sent for diagnostic purposes to the clinical microbiology laboratory of our university hospital (AP-HM, located at the Institut Hospitalo-Universitaire Méditerranée Infection, Marseille, France) were retrospectively cultivated using a culturomics method as allowed by French law (Article L.1211-2 of the Public Health Code). Patients had been informed of the possible reuse for research purposes of their samples and their personal data collected during care. They had the possibility of opposing this by reporting it to the Data Protection Officer of the AP-HM. Neither patient had expressed any objection. The patient personal data used for analysis were anonymised. Our independent local ethics committee approved (Agreement Nos. 2022-005 and 2022-009) the clearance of Ethics Review Committee and compliance with data protection legislation.

Strain Marseille-Q7072^T^ was isolated from a vaginal sample taken from a 41-year-old woman who had suffered from recurrent miscarriages. Strain Marseille-Q7826^T^ was isolated from a vaginal sample taken from a 30-year-old pregnant woman who was at 26 weeks of amenorrhoea. Neither patient had bacterial vaginosis or a sexually transmitted infection at the time of the sampling.

### Strain Isolation

Two different strains, Marseille-Q7072^T^ and Marseille-Q7826^T^, were cultured from vaginal samples using different pre-incubation methods. For Marseille-Q7072^T^, a vaginal sample was pre-incubated at 37 °C in anaerobic blood culture vials (Becton Dickinson, Le Pont-de-Claix, France) supplemented with 40 ml of Tryptic Soy Broth (Sigma-Aldrich, St. Louis, USA) for seven days. For Marseille-Q7826^T^, a vaginal sample was pre-incubated at 37 °C in anaerobic blood culture vials (Becton Dickinson) supplemented with 40 ml of Difco Marine Broth (Becton Dickinson) for 30 days. Subsequently, isolated colonies were subcultured on Columbia agar with 5% sheep blood (bioMérieux, Marcy l’Etoile, France) at 37 °C under anaerobic conditions using AnaeroGen (bioMérieux) after 48 h.

### MALDI-TOF Identification

A Microflex LT MALDI-TOF mass spectrometer (Bruker Daltonics, Bremen, Germany) [6] was used to identify Marseille-Q7072^T^ and Marseille-Q7826^T^. The MALDI BioTyper software (version 2.0, Bruker) was used to import and analyse the spectra of the two strains. Standard pattern matching was employed with default parameter settings, and the resulting scores were interpreted as described previously [7].

### Morphological Observation and Phenotypic Characterisation

By culturing each strain under different atmospheres, temperatures, pH, as well as salinity parameters, the optimum growth conditions for the two strains were identified. Different growth temperatures (room temperature, 28 °C, 37 °C, 42 °C, and 56 °C), various pH conditions ranging from 5.5 to 8.5, as well as different levels of salinity, including 0%, 5%, 7.5%, 10%, 15%, and 20%, were tested on Columbia agar with 5% sheep blood (bioMérieux) under aerobic, anaerobic (GENbag anaer, bioMérieux), and microaerophilic (GENbag Microaer, bioMérieux) conditions.

The phenotypic characteristics of the two strains including Gram staining, motility, sporulation, oxidase, and catalase activities were tested, as preliminarily reported by Ly and colleagues [8]. In addition, their morphology was observed using a SU5000 scanning electron microscope (SEM, Hitachi High-Tech, Tokyo, Japan), as similarly described by Zgheib et al*.* [9].

For the biochemical properties, three API gallery systems (API® ZYM, API® 20A, and API® 50 CH [bioMérieux]) were evaluated according to the manufacturer’s instructions. E-test gradient strips (bioMérieux) were used to determine antibiotic susceptibility according to EUCAST recommendations [10]. Finally, analysis of cellular fatty acid methyl esters (FAME) was conducted by gas chromatography/mass spectrometry (GC/MS), as previously reported [11, 12].

### Genome Extraction, Sequencing, Annotation, and Comparison

Mechanical lysis was performed on the two strains with acid-washed glass beads (G4649-500 g, Sigma-Aldrich) by a FastPrep BIO 101 instrument (Qbiogene, Strasbourg, France) at maximum speed (6.5 m/s) for 90 s, followed by a two-hour lysozyme incubation at 37 °C. DNA extraction was then performed using the EZ1 instrument and the EZ1 DNA Tissue kit (Qiagen, Hilden, Germany).

The DNA extract of both strains was sequenced using a MiSeq sequencer (Illumina Inc., San Diego, CA, USA) via the Nextera Mate Pair sample preparation kit and the Nextera XT Paired End (Illumina), as previously mentioned [13]. SPAdes 3.13.1 software was used at default settings to assemble the reads [14]. Scaffolds with nucleotide counts < 800 bp and scaffolds with depth values < 25% of the average depths were suppressed. The resulting genome for each strain and the genomes of the closely related species were annotated with Prokka 1.14.5, as described previously [15, 16], and compared. The 16S rRNA sequences of the two strains were extracted from their respective genomes and then blasted using BLASTn NCBI. The Clusters of Orthologous Groups (COG) database was used for bacterial protein sequences detection using BLASTP [17].

To evaluate the overall similarity between the compared genomes [18, 19], digital DNA-DNA hybridisation (dDDH) was carried out using the Genome-to-Genome Distance Calculator (GGDC) 2.1 web server (http://ggdc.dsmz.de/distcalc2.php). The average amino acid identity (AAI) calculation between the two paired strains was obtained through the CompareM packages (https://github.com/dparks1134/CompareM). Average nucleotide identity (ANI) analysis was attempted using the OrthoANI 1.2 software [20]. The species thresholds for dDDH and AAI/ANI are 70% and 95–96%, respectively [19, 21].

Furthermore, a phylogenetic tree was constructed from seven concatenated genes, namely *dnaA*, *gyrA*, *gyrB*, *recA*, *rpoB*, *rplE*, and *rplD*. This tree was built using the Iqtree software (v1.6.12) [22] and employed the maximum-likelihood method for phylogenetic inference. Subsequently, the sequences were aligned using MAFFT [23]. Bootstrap values, derived from 1000 iterations of the analysis to create a majority consensus tree, are indicated at nodes [24]. Only bootstrap values exceeding 70% are presented. Genomic sequences from various species under investigation were obtained from the NCBI database. These sequences were then annotated using the Prokka software [15]. The target genes were extracted from the genomes and concatenated using the Emboss union server (https://www.bioinformatics.nl/cgi-bin/emboss/union).

## Results

### Strain Identification and Phylogenetic Analysis

**Fig. 1:**
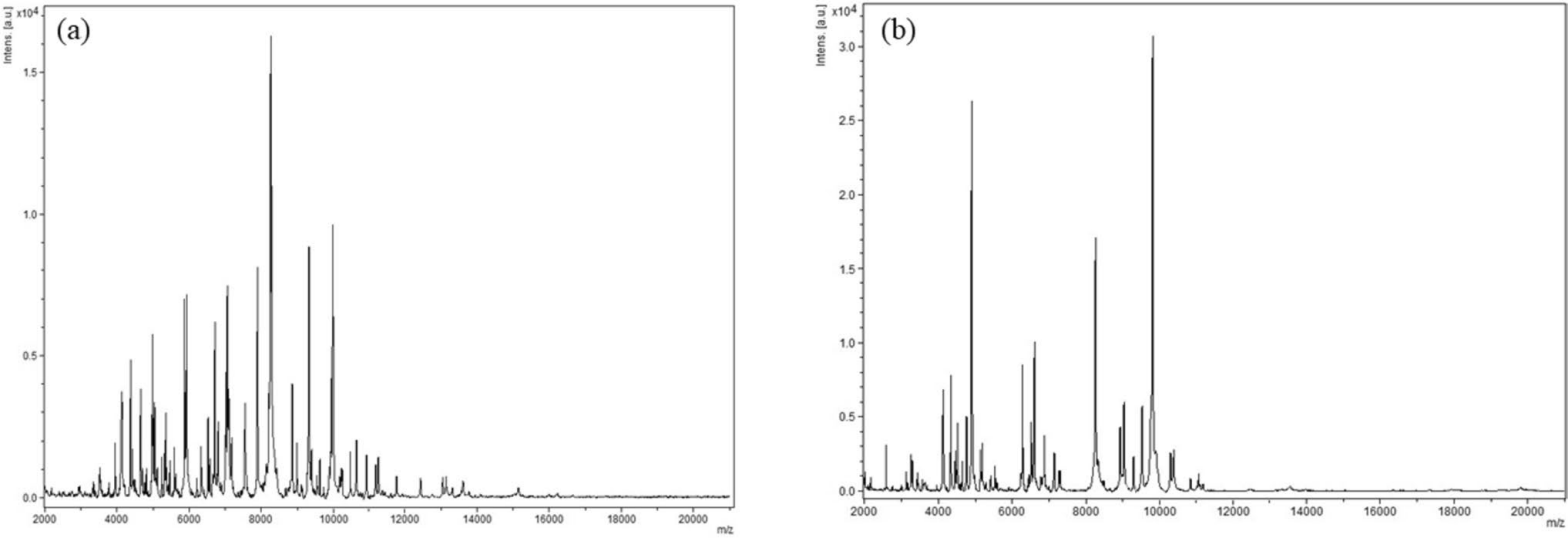
MALDI-TOF MS reference spectra of **a** *Peptinophilus genitalis* sp. nov., strain Marseille-Q7072^T^ and **b** *Mobiluncus massiliensis* sp. nov., strain Marseille-Q7826^T^. The reference spectrum was generated by comparison of spectra from 6 individual colonies using the Biotyper 3.0 software

Strains Marseille-Q7072^T^ and Marseille-Q7826^T^ were isolated from human vaginal samples. Systematic analysis using MALDI-TOF mass spectrometry failed to identify the two strains (Fig. 1). For both strains, the scores were lower than 1.8, indicating that the corresponding species were not found in the database and that they could potentially be an unknown species.

Strains Marseille-Q7072^T^ and Marseille-Q7826^T^ had 16S rRNA sequences that shared 99% similarity with *Peptoniphilus harei* strain NCTN13077 (LR134524.1) and 99.5% similarity with *Mobiluncus curtisii* strain ATCC 35241 T (GL385912.1), respectively. However, the dDDH values for the Marseille-Q7072^T^ and Marseille-Q7826^T^ strains were highest, respectively, with *P. coxii* (46.9%) and *M. curtisii* (29.5%), two bacterial species listed on the website of List of Prokaryotic names with Standing in Nomenclature (LPSN) (https://lpsn.dsmz.de/, accessed 22 November 2023), but remained below the threshold used to distinguish prokaryotic species (70%). Similarly, the highest OrthoANI values of the Marseille-Q7072^T^ and Marseille-Q7826^T^ strains were 88% with *P. harei* and 86% with *M. curtisii*, respectively, but were below the 95% threshold.

Based on these results, strains Marseille-Q7072^T^ and Marseille-Q7826^T^ were considered to be representative of two putative new species within the family *Peptoniphilaceae* in the phylum *Bacillota* and within the family *Actinomycetaceae* in the phylum *Actinomycetota,* respectively.

### Phenotypic Characterisation

Optimal growth of Marseille-Q7072^T^ and Marseille-Q7826^T^ strains was obtained after two days of culture at 37 °C under anaerobic conditions. The main characteristics of the two strains are presented in Supplementary Table 1.

**Fig. 2:**
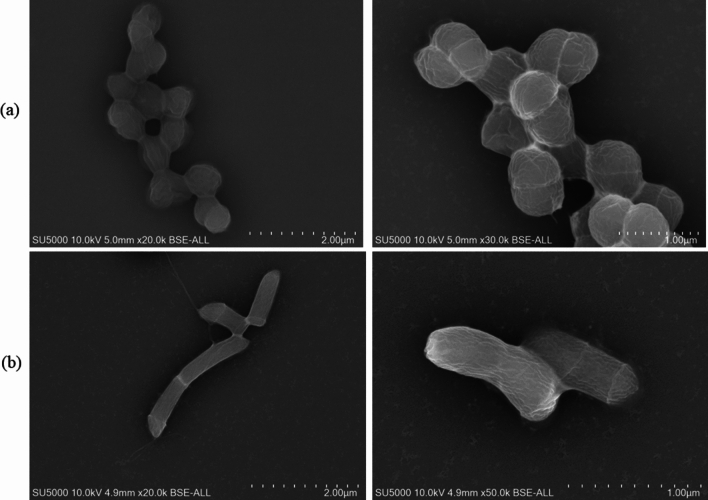
Scanning electron microscopy of **a** *Peptoniphilus genitalis* sp. nov., strain Marseille-Q7072^T^ and **b** *Mobiluncus massiliensis* sp. nov., strain Marseille-Q7826^T^

Strain Marseille-Q7072^T^ is an anaerobic and microaerophilic, Gram-stain-positive, non-spore-forming, non-motile, and coccus-shaped bacterium. Growth occurs under an anaerobic atmosphere within a temperature range of between 20 and 37 °C (optimum 37 °C), at pH 6–8.5 (optimum pH 7), and with 0–5% (w/v) NaCl (best < 5%), but also under microaerophilic conditions. Under optimal growing conditions, colonies appear circular, grey, opaque, and convex with a diameter of 2–3 mm. Catalase and oxidase activities are negative. As observed using electron microscopy, bacterial cells have a diameter of 0.609 μm ± 0.087 μm and are arranged in clusters (Fig. 2a).

Strain Marseille-Q7826^T^ is a facultative anaerobic, Gram-stain-variable, non-spore-forming, motile, and curved rod-shaped bacterium. Growth occurs under an anaerobic atmosphere in a temperature range of between 20 and 37 °C (optimum 37 °C), at pH 6–7.5 (optimum pH 7) and without adding NaCl. Under optimal growing conditions, colonies appear circular, colourless, translucent, and convex, with a diameter of 2–3 mm. Catalase and oxidase activities are negative. In electron microscopy, bacterial cells have a length of 1.216 μm ± 0.243 μm and a diameter of 0.381 μm ± 0.039 μm (Fig. 2b).

For strain Marseille-Q7072^T^, using an API ZYM strip, positive reactions were observed for leucine arylamidase, naphtol-AS-BI-phosphohydrolase, and acid phosphatase. Using an API 20A strip, strain Marseille-Q7072^T^ exhibited positive reactions for gelatine, esculin, d-trehalose, and D-glucose. Moreover, using API 50 CH strip, strain Marseille-Q7072^T^ was positive for esculin, salicin, lactose, melezitose, xylitol, L-fucose, L-arabitol, and 5-keto-gluconate. These results were compared to those of *Peptoniphilus coxii* RMA 16757 [25], *Peptoniphilus harei* DSM 10020 [26], and *Peptoniphilus timonensis* JC401 [27] (Table 1).

**Table 1:** Comparison of *Peptoniphilus genitalis* sp. nov., strain Marseille-Q7072^T^ and *Mobiluncus massiliensis* sp. nov., strain Marseille-Q7826^T^ with their phylogenetically closest species with a validly published name

Properties	Pgen	Pcox	Pharei	Ptim	Mobmass	Mobcurt	Mobhom	Mobmul
Cell size	0.6 µm	< 0.7 µm	0.5–1.5 µm	0.91 µm	1.2 µm	1.7 µm	1.7 µm	2.9 µm
0_2_ requirement	Anaerobic and microaerophilic	Anaerobic	Anaerobic	Anaerobic	Anaerobic	Anaerobic	Anaerobic	Anaerobic
Gram stain	+	+	+	+	Variable	Variable	Variable	Variable
Mobility	−	−	−	−	+	+	−	+
Catalase	−	−	−	+	−	−	−	−
Production of:								
Acid phosphatase	+	NA	−	−	+	w	NA	w
Alkaline phosphatase	−	−	−	−	−	w	w	NA
NAphthol-AS-BI-phosphohydrolase	+	NA	−	−	−	NA	NA	NA
Valine arylamidase	−	NA	−	−	−	NA	NA	NA
α-glucosidase	−	−	−	−	+	+	w	+
ß-glucosidase	−	−	−	−	+	−	w	−
ß-glucuronidase	−	−	−	−	−	−	NA	−
Utilization of:				−				
D-glucose	+	NA	−	−	+	w	−	w
D-sucrose	−	NA	−	−	−	−	−	−
D-mannose	−	+	−	−	+	−	−	−
G + C content (mol%)	34.3	44.62	34.4	30.7	57	51–52	NA	49–50
Habitat	Human vagina	Human specimens	Human sacral ulcer	Human stool	Human vagina	Human vagina	Human vagina	Human vagina

Strains: *Pgen* *Peptoniphilus genitalis* sp. nov., strain Marseille-Q7072; *Pcox* *Peptoniphilus coxii* RMA 16757 [25]; *Pharei* *Peptoniphilus harei* DSM 10020 [26]*;* *Ptim* *Peptoniphilus timonensis* JC401 [27]*;* *Mobmass* *Mobiluncus massiliensis* sp. nov., strain Marseille-Q7826; *Mobcurt* *Mobiluncus curtisii* BV345-16 [28]; *Mobhom* *Mobiluncus homelsii* BV376-6 [28]; *Mobmul*, *Mobiluncus mulieris* BV64-5 [28]

+  Positive,  − Negative, *w* Weakly positive, *NA* Not available

For strain Marseille-Q7826^T^, using an API ZYM strip, positive reactions were detected for leucine arylamidase, naphtol-AS-BI-phosphohydrolase, ß-galactosidase, D-glucosidase, esterase, phosphatase acid, ß-glucosidase, and D-mannosidase. Using an API 20A strip, strain Marseille-Q7826^T^ exhibited positive reactions for D-glucose, D-lactose, D-maltose, salicin, D-xylose, esculin, and D-mannose. Finally, using an API 50 CH strip, strain Marseille-Q7826^T^ was positive for D-xylose, esculin, starch, gluconate, and 5-ketogluconate. These results were compared to those of *Mobiluncus curtisii* BV345-16 [28], *Mobiluncus homelsii* BV376-6 [28] and *Mobiluncus mulieris* BV64-5 [28] (Table 1).

For strain Marseille-Q7072^T^, the most abundant fatty acid was C16:0 (46%), followed by C18:1n9 (15%), and C14:0 (11%). For strain Marseille-Q7826^T^, the most abundant fatty acid was C16:0 (54%), followed by C18:1n9 (18%), and C18:2n6 (15%). Minor amounts of unsaturated, branched, and saturated fatty acids were also described (Table 2).

**Table 2:** Cellular fatty acids composition (%) of strains Marseille-Q7072^T^ (*Peptoniphilus genitalis* sp. nov.)*, Peptoniphilus harei* DSM 10020, Marseille-Q7826^T^ (*Mobiluncus massiliensis* sp. nov.)*,* and *Mobiluncus curtisii* BV345-16

Fatty acids	Name	*Peptoniphilus genitalis* sp. nov., Marseille-Q7072	*Peptoniphilus harei* DSM 10020	*Mobiluncus massiliensis* sp. nov., Marseille-Q7826	*Mobiluncus curtisii* BV345-16^a^
C_16:0_	Hexadecanoic acid	46.1	32.1	54.0	38.2
C_18:1n9_	9-octadecenoic acid	15.6	17.0	18.6	18.8
C_14:0_	Tetradecanoic acid	11.5	4.4	0.8	2.1
C_18:2n6_	9,12-octadecadienoic acid	10.6	17.0	15.1	20.9
C_18:0_	Octadecanoic acid	6.6	7.2	6.4	15.7
C_16:1n7_	9‐Hexadecenoic acid	6.0	1.0	ND	2.1
C_18:1n7_	11‐Octadecenoic acid	1.9	1.9	TR	ND
C_15:0_	Pentadecanoic acid	1.1	ND	TR	ND
C_17:0_	Heptadecanoic acid	TR	ND	TR	1.3
C_17:0 anteiso_	14‐Methyl‐hexadecanoic acid	ND	4.2	ND	1.3
C_14:0_	Tetradecanoic acid	ND	4.4	TR	ND
C_17:1n7_	10-Heptadecenoic acid	ND	ND	4.1	ND

*TR* trace amounts < 1%, *ND* Not detected

^a^Percentage values were corrected from source by removing non fatty acid structures (DMA and unknown

The antimicrobial susceptibilities of Marseille-Q7072^T^ and Marseille-Q7826^T^ strains were evaluated against various antibiotics. For the strain Marseille-Q7072^T^, the minimum inhibitory concentrations were as follows: < 0.016 μg/mL for penicillin G, 0.023 μg/mL for amoxicillin, 0.47 μg/mL for ceftriaxone, 0.19 μg/mL for ceftazidime, 0.004 μg/mL for imipenem, 1.5 μg/mL for ciprofloxacin, 0.5 μg/mL for azithromycin, 0.5 μg/mL for clindamycin, 0.016 μg/mL for daptomycin, 1.5 μg/mL for doxycycline, 8 μg/mL for fosfomycin, 1.5 μg/mL for gentamicin, 24 μg/mL for tobramycin, 0.5 μg/mL for metronidazole, 0.75 μg/mL for nitrofurantoin, 0.002 μg/mL for rifampicin, 0.75 μg/mL for linezolid, < 0.016 μg/mL for teicoplanin, and 0.064 μg/mL for vancomycin. Amikacin and trimethoprim-sulfamethoxazole showed no activity against strain Marseille-Q7072^T^.

For the strain Marseille-Q7826^T^, the minimum inhibitory concentrations were as follows: 0.16 μg/mL for penicillin G, 0.016 μg/mL for amoxicillin, 0.75 μg/mL for ceftriaxone, 0.38 μg/mL for ceftazidime, 0.002 μg/mL for imipenem, < 0.016 μg/mL for clindamycin, 0.38 μg/mL for azithromycin, 0.19 μg/mL for daptomycin, 8 μg/mL for doxycycline, 0.094 μg/mL for gentamicin, 1.5 μg/mL for amikacin, 0.25 μg/mL for tobramycin, 0.0125 μg/mL for nitrofurantoin, < 0.002 μg/mL for rifampicin, 0.19 μg/mL for linezolid, 0.023 μg/mL for teicoplanin, and 0.125 μg/mL for vancomycin. Ciprofloxacin, fosfomycin, and metronidazole showed no activity against strain Marseille-Q7826^T^.

### Genomic Analysis

**Fig. 3:**
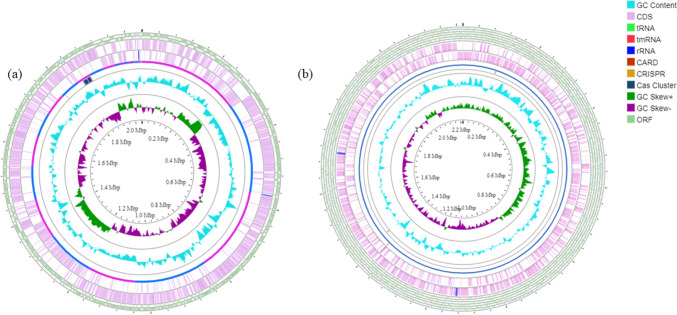
Graphical circular map of genomes of **a** *Peptoniphilus genitalis* sp. nov., strain Marseille-Q7072^T^ and **b** *Mobiluncus massiliensis* sp. nov., strain Marseille-Q7826^T^

For strain Marseille-Q7072^T^, the number of reads was 879 694. The genome length was 2 040 803 bp, assembled into 33 contigs, with a G + C content of 34.3 mol% (Fig. 3a). Strain Marseille-Q7072^T^ had 1947 predicted genes, including 1911 protein-coding genes. Strain Marseille-Q7072^T^ also had 36 RNA-coding genes, including four rRNA, 31 tRNA, and one tmRNA.

For strain Marseille-Q7826^T^, the number of reads was 1 732 034. The genome length was 2 216 029 bp, assembled into one contig, with a G + C content of 57 mol% (Fig. 3b). Strain Marseille-Q7826^T^ had 1908 predicted genes, including 1855 protein-coding genes. Strain Marseille-Q7826^T^ also had 53 RNA-coding genes, including six rRNA, 46 tRNA, and one tmRNA.

**Fig. 4:**
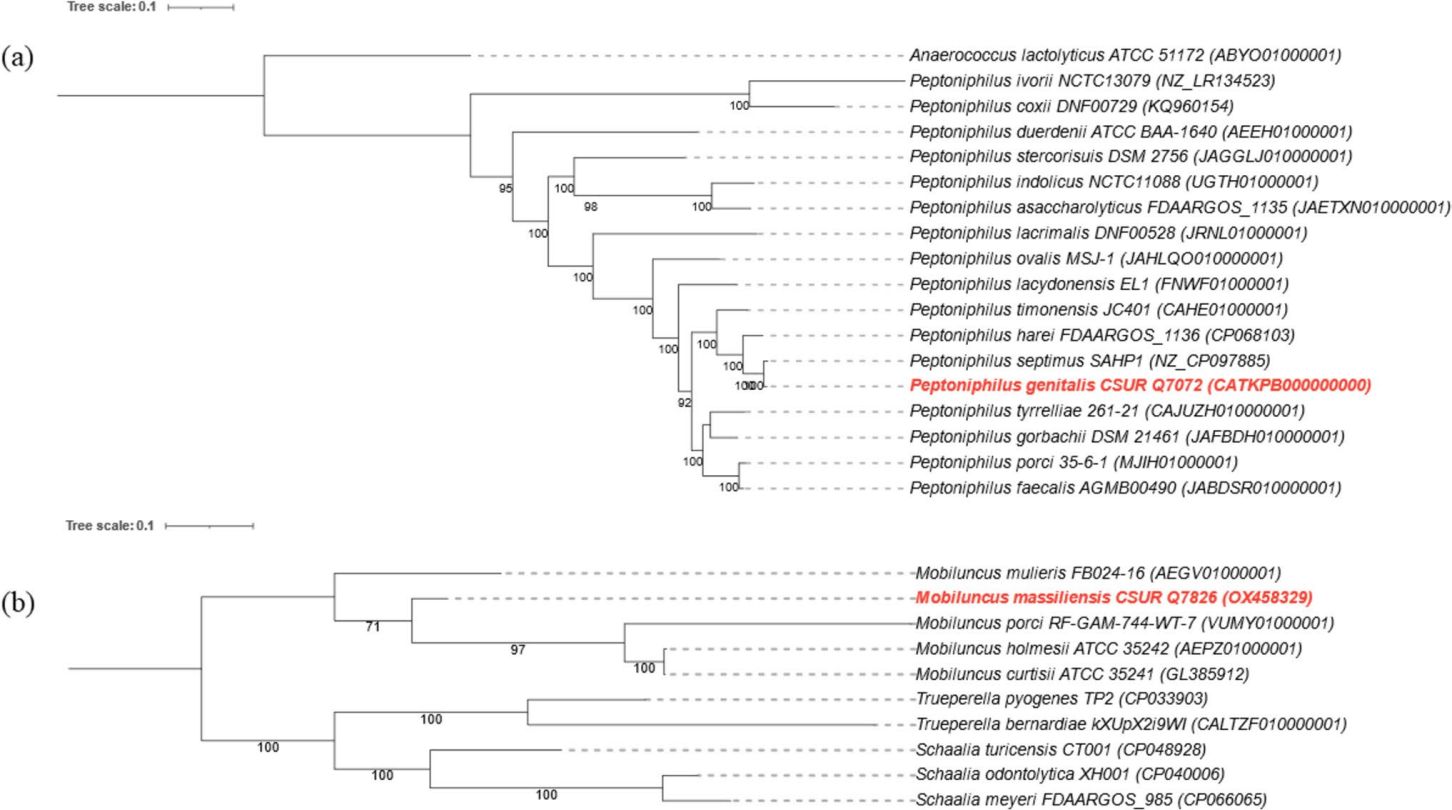
Phylogenetic trees based on concatened sequences of 7 housekeeping genes **a** *Peptoniphilus genitalis* sp. nov., strain Marseille-Q7072^T^ and **b** *Mobiluncus massiliensis* sp. nov., strain Marseille-Q7826^T^ (Bold), and closely related species

The distribution of functional classes of predicted genes for strains Marseille-Q7072^T^ and Marseille-Q7826^T^ strains according to the COGs database is also presented in Table 3. Figure 4 shows two phylogenetic trees based on concatened sequences of 7 housekeeping genes, highlighting the position of each strain in relation to other closely related species. Strain Marseille-Q7072^T^ is most closely related to “*Peptoniphilus septimus*” SAHP1, a bacterium recently reported in a case of blood mono-infection in a cervical cancer patient receiving chemotherapy, not well described, not available in a strain collection and not officially recognized by nomenclature [29] and *P. harei* FDAARGOS_1136 within the genus *Peptoniphilus*.

**Table 3:** Number of genes associated with the 25 general COG functional categories of *Peptoniphilus genitalis* sp. nov., strain Marseille-Q7072^T^ and *Mobiluncus massiliensis* sp. nov., strain Marseille-Q7826^T^

Code	*Peptoniphilus genitalis*		*Mobiluncus massiliensis*		Description
	Value	% of total	Value	% of total	
[J]	136	7.12	132	7.12	Translation
[A]	0	0	1	0.05	Rna processing and modification
[K]	96	5.02	61	3.29	Transcription
[L]	99	5.18	89	4.80	Replication, recombination and repair
[B]	0	0	0	0	Chromatin structure and dynamics
[D]	19	0.99	22	1.19	Cell cycle control, mitosis and meiosis
[Y]	0	0	0	0	Nuclear structure
[V]	72	3.77	40	2.16	Defense mechanisms
[T]	36	1.88	35	1.89	Signal transduction mechanisms
[M]	56	2.93	85	4.58	Cell wall/membrane biogenesis
[N]	0	0	15	0.81	Cell motility
[Z]	0	0	0	0	Cytoskeleton
[W]	0	0	0	0	Extracellular structures
[U]	17	0.89	27	1.46	Intracellular trafficking and secretion
[O]	50	2.62	53	2.86	Posttanslational modification, protein turnover,chaperones
[X]	0	0	0	0	Mobilome: prophages, transposons
[C]	84	4.40	87	4.69	Energy production and conversion
[G]	32	1.67	103	5.55	Carbohydrate transport and metabolism
[E]	103	5.39	102	5.50	Amino acid transport and metabolism
[F]	48	2.51	51	2.75	Nucleotide transport and metabolism
[H]	46	2.41	59	3.18	Coenzyme transport and metabolism
[I]	39	2.04	22	1.19	Lipid transport and metabolism
[P]	92	4.81	53	2.86	Inorganic ion transport and metabolism
[Q]	13	0.68	5	0.27	Secondary metabolites biosynthesis, transport and catabolism
[R]	157	8.22	137	7.39	General function prediction only
[S]	131	6.86	90	4.85	Function unknown
–	99	5.18	157	8.46	Not in COGs

Strain Marseille-Q7826^T^ is most closely related to *M. curtisii* ATCC 35241^ T^ and *M. holmesii* ATCC 35242^ T^ within the genus *Mobiluncus*.

**Fig. 5:**
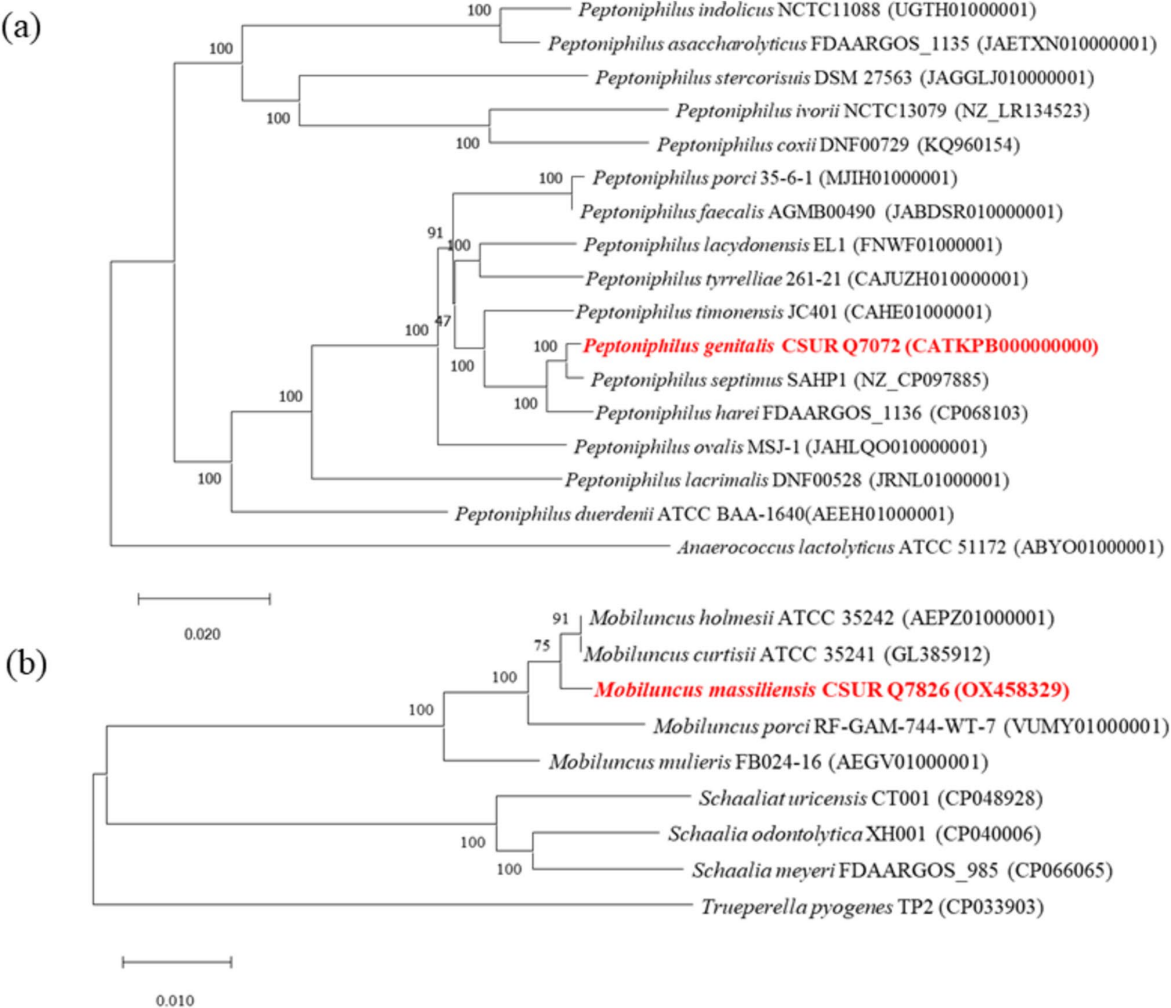
Phylogenetic tree based on 16S rRNA of **a** *Peptoniphilus genitalis* sp. nov., strain Marseille-Q7072^T^ and **b** *Mobiluncus massiliensis* sp. nov., strain Marseille-Q7826^T^ (Bold), and closely related species

Furthermore, the genomic features of these two new strains are statistically compared with other related species (Table 4). For strain Marseille-Q7072^T^, if we include in the analysis exclusively bacterial species currently validated by the nomenclature, the highest dDDH value is 46.9% with *P. coxii* (Table 5), the OrthoANI values ranged from 69 to 88% (Supplementary Fig. 1) and the highest AAI value is 88% with *P. harei* (Supplementary Table 2). These estimates are therefore below the thresholds required to distinguish prokaryotic species (dDDH < 70% and AAI/ANI < 95 ~ 96%), indicating that strain Marseille-Q7072^T^ is a new bacterial species. However, if we include in the analysis all sequences available in current databases, the highest dDDH value is 69.3% with “*P. septimus*” (Table 5) and the highest AAI value is 95.94% with “*P. septimus*” (Supplementary Table 2). For strain Marseille-Q7826^T^, the highest dDDH value is 29.5% with *M. curtisii* (Table 5), the OrthoANI values ranged from 71 to 86% (Supplementary Fig. 2) and the highest AAI value is 89.24% with *M. holmesii* (Supplementary Table 2), confirming that this strain is different from other bacterial strains. It should also be noted that the Marseille-Q7072^T^ strain showed 16S rRNA sequence similarities of 99.67% with the “*P. septimus*” SAHP1 strain (CP097885.1) and 99% with the *P. harei* NCTN13077 strain (LR134524.1) (Fig. 5). The Marseille-Q7826^T^ strain showed 99.54% 16S rRNA sequence similarity with *M. curtisii* ATCC 35241 T (GL385912.1), the closest species phylogenetically (Fig. 5). These identity values were therefore above the threshold of 98.65% for the definition of a new bacterial species [21, 30]. However, it has now been clearly demonstrated that the thresholds, originally designed to standardise the use of 16S rRNA gene sequences in taxonomy, do not apply to several genera [31].

**Table 4:** Summary of genome properties for *Peptoniphilus genitalis* sp. nov., strain Marseille-Q7072^T^, *Mobiluncus massiliensis* sp. nov., strain Marseille-Q7826^T^ compared with their phylogenetically closest species with a validly published name

Strains	Accession	Size (bp)	G + C (%)	Total genes	Protein-coding genes	rRNAs	tRNAs
Pgen	CATKPB000000000	2,040,803	34.3	1,947	1,911	4	31
Pharei	CP068103	1,929,566	33.9	1,828	1,777	9	41
Mobmass	OX458329	2,216,029	57.0	1,908	1,855	6	46
Mobcurt	GL385912	2,137,068	55.3	1,871	1,823	0	47

Strains: *Pgen* *Peptinophilus genitalis* Marseille-Q7072, *Pharei* *Peptoniphilus harei* FDAARGOS_1136, *Mobmass* *Mobiluncus massiliensis* Marseille-Q7826, *Mobcurt* *Mobiluncus curtisii* ATCC 35241

**Table 5:** dDDH values of *Peptoniphilus genitalis* sp. nov., strain Marseille-Q7072^T^ and *Mobiluncus massiliensis* sp. nov., strain Marseille-Q7826^T^ with other closely related species with standing in nomenclature

Query strain	Subject strain	dDDH (in %)	G + C content difference (in %)
	*“Peptoniphilus septimus”*	69.3	0.32
Q7072	*Peptoniphilus coxii*	46.9	10.48
	*Peptoniphilus harei*	35.1	0.4
	*Peptoniphilus ivorii*	33.5	18.96
	*Peptoniphilus asaccharolyticus*	30.2	1.87
	*Peptoniphilus lacrimalis*	29.2	0.49
	*Peptoniphilus duerdenii*	28.5	0.02
	*Peptoniphilus indolicus*	28.2	2.68
	*Peptoniphilus timonensis*	26.1	3.58
	*Peptoniphilus gorbachii*	24.8	2.86
	*Peptoniphilus tyrrelliae*	24.3	2.23
	*Peptoniphilus porci*	22.6	3.06
	*Peptoniphilus lacydonensis*	22	4.36
	*Peptoniphilus faecalis*	21.9	3.85
	*Peptoniphilus ovalis*	20	3.61
	*Peptoniphilus stercorisuis*	17.2	6.39
Q7826	*Schaalia turicensis*	30	0.1
	*Mobiluncus curtisii*	29.5	1.37
	*Mobiluncus holmesii*	29.4	1.4
	*Mobiluncus mulieris*	27.3	1.96
	*Schaalia meyeri*	26.9	8.55
	*Trueperella pyogenes*	24.5	2.67
	*Schaalia odontolytica*	24.1	8.89
	*Mobiluncus porci*	20.5	0.73
	*Trueperella bernardiae*	19.3	9.19

Taken as a whole, the above information supports the proposals that strain Marseille-Q7826^T^ represents a new species in the family *Actinomycetaceae*, for which the name *Mobiluncus massiliensis* sp. nov. is proposed. Strain Marseille-Q7072^T^ represents a species in the family *Peptoniphilaceae.* Genomic analyses indicate that it corresponds to “*P. septimus*” SAHP1, a strain that has not been accurately described, is not available in any strain collection and has therefore not been officially validated within ICNP. Thus, the proposed name for this species is *Peptoniphilus genitalis* sp. nov. and “*Peptoniphilus septimus*” SAHP1 is an earlier synonym.

## Discussion

The cut-off values used to classify bacterial strains at the genus and species level, established under the assumption that the level of 16S rRNA sequence variation between species was homogeneous among genera, proved inadequate for many genera. For example, Rossi-Tamisier et al*.* have shown that in only 17 of the 158 genera in their study (10.8%), all species respected the 95% and 98.7% thresholds for genus and species levels, respectively [31]. Thus, given the limitations of using 16S rRNA sequence analysis to classify bacterial species, whole genome sequencing remains the more advanced and accurate strategy to determining the taxonogenomic identity of a bacterium.

Strain Marseille-Q7072^T^ is a new member of *Bacillota,* for which the name *Peptoniphilus genitalis* sp. nov. is proposed. Currently, the genus *Peptoniphilus* contains 21 species with validly published names and thirteen species without validly published names (https://lpsn.dsmz.de/search?word=Peptoniphilus, accessed 22 November 2023). Growth is mainly strict anaerobic but some bacteria of the genus *Peptoniphilus* such as *Peptoniphilus genitalis* sp. nov., *Peptoniphilus lacydonensis*, and *Peptoniphilus nemausensis* have the ability to grow under microaerophilic conditions [32]. Most species of *Peptoniphilus* have been detected in human clinical specimens including the vagina [26, 33, 34]. They are part of the human commensal flora, but they are also opportunistic pathogens, particularly in the context of polymicrobial infections [33–35].

Strain Marseille-Q7826^T^ is a new member of *Actinomycetota,* for which the name *Mobiluncus massiliensis* sp. nov. is proposed. The genus *Mobiluncus* is currently composed of four species: *Mobiluncus curtisii*, *Mobiluncus holmesii*, *Mobiluncus mulieris*, and *Mobiluncus porci* (https://lpsn.dsmz.de/search?word=Mobiluncus, accessed 22 November 2023). With the exception of *M. porci*, which was isolated from pig intestines [36], all other species have most often been isolated from human genital sites, mainly the vagina [37], and especially in cases of bacterial vaginosis. However, their role in bacterial vaginosis remains poorly understood and unclear.

## Conclusion

In conclusion, based on the results of phenotypic, phylogenetic and genomic analyses, and taking into account only bacterial species validated according to nomenclature, strains Marseille-Q7072^T^ and Marseille-Q7826^T^ represent new bacterial species. If we take into account all the sequences available in the databases, the genomic data for Marseille-Q7072^T^ indicate that it corresponds to “*P. septimus*” SAHP1. However, as the strain of “*P. septimus*” SAHP1 is not available, it is not possible to go any further in comparing these two strains. These data underline the importance of at least systematically depositing every new bacterial strain in a collection, in order to make it accessible to the scientific community.

### Description of *Peptoniphilus genitalis *sp. nov. (Earlier synonym “*Peptinophilus septimus*”)

*Peptoniphilus genitalis* (ge.ni.ta’lis. L. masc. adj. *genitalis*, belonging to the genital system). Cells are anaerobic and microaerophilic, Gram-stain-positive, non-spore-forming, non-motile, and coccus-shaped. Bacterial cells are 0.609 μm ± 0.087 μm in diameter and positioned in clusters. Catalase and oxidase activities are negative. After 48 h of incubation on Columbia agar with 5% sheep blood, colonies appear circular, grey, opaque, and convex, with a diameter of between 2 and 3 mm. Growth occurs under an anaerobic atmosphere in a temperature range of 20–37 °C (optimum 37 °C), at pH 6–8.5 (optimum pH 7) and with 0%–5% (w/v) NaCl (optimum < 5%).

Using API ZYM, 20A, and 50 CH strips, positive reactions were obtained for leucine arylamidase, Naphtol-AS-BI-phosphohydrolase, acid phosphatase, gelatine, esculin, D-trehalose, D-glucose, salicin, lactose, melezitose, xylitol, L-fucose, L-arabitol, and 5-keto-gluconate.

The most abundant fatty acid was C_16:0_ followed by C_18:1n9_ and C_14:0_. The size of the genome is 2.04 Mbp and its G + C content is 34.3 mol%.

The type strain Marseille-Q7072^T^ (= CSUR Q7072^T^ = CECT 30604^ T^) was isolated from a vaginal sample of a 41-year-old woman who had suffered recurrent miscarriages.

The 16S rRNA and genome sequences were deposited in GenBank under accession numbers OX458314 and CATKPB000000000, respectively.

### Description of *Mobiluncus massiliensis* sp. nov.

*Mobiluncus massiliensis* (mas.si.li.en’sis. N.L. masc. adj. *massiliensis*, from “Massilia”, the Latin name of Marseille).

Cells are facultative anaerobic, Gram-stain-variable, non-spore-forming, motile, and curved rod-shaped. Bacterial cells have a length of 1.216 μm ± 0.243 μm and a diameter of 0.381 μm ± 0.039 μm. Catalase and oxidase activities are negative. After 48 h of incubation on Columbia agar with 5% sheep blood, colonies appear circular, colourless, translucent and convex, with a diameter of between 2 and 3 mm. Growth occurs under an anaerobic atmosphere in a temperature range of between 20 and 37 °C (optimum 37 °C), at pH 6–7.5 (optimum pH 7) and without adding NaCl.

Using API ZYM, 20A, and 50 CH strips, positive reactions were obtained for leucine arylamidase, naphtol-AS-BI-phosphohydrolase, ß-galactosidase, D-glucosidase, esterase, phosphatase acid, ß-glucosidase, D-mannosidase, D-glucose, D-lactose, D-maltose, salicin, D-xylose, esculin, D-mannose, starch, gluconate, and 5-ketogluconate.

The most abundant fatty acid by far was C_16:0_ followed by C_18:1n9_ and C_18:2n6_. The size of the genome is 2.21 Mbp and its G + C content is 57 mol%.

The type strain Marseille-Q7826^T^ (= CSUR Q7826^T^ = CECT 30727^ T^) was isolated from a vaginal sample taken from a 30-year-old pregnant woman at 26 weeks of amenorrhoea.

The 16S rRNA and genome sequences were deposited in GenBank under accession numbers OX458307 and OX458329, respectively.

### Supplementary Information

Below is the link to the electronic supplementary material.

Supplementary file1 (TIF 145 KB)—Heat map generated with OrthoANI values between Peptoniphilus genitalis sp. nov., strain Marseille-Q7072T and other closely related species with standing in the nomenclature.

Supplementary file2 (TIF 87 KB)—Heat map generated with OrthoANI values between Mobiluncus massiliensis sp. nov., strain Marseille-Q7826T and other closely related species with standing in the nomenclature.

Supplementary file3 (DOCX 21 KB)

## Data Availability

The datasets presented in this search are available in online repositories. The names of the repository(s) and accession number(s) can be found below: https://www.ncbi.nlm.nih.gov/nuccore/CATKPB00000000; https://www.ncbi.nlm.nih.gov/nuccore/OX458314; https://www.ncbi.nlm.nih.gov/nuccore/OX458329; https://www.ncbi.nlm.nih.gov/nuccore/OX458307
