# Supplementary figures and images for: Peptoniphilus genitalis sp. nov. and Mobiluncus massiliensis sp. nov.: Novel Bacteria Isolated from the Vaginal Microbiome
Source: Curr Microbiol. 2024 Feb 19;81(4):97. doi: 10.1007/s00284-023-03584-7 (PMC10876752; doi:10.1007/s00284-023-03584-7)

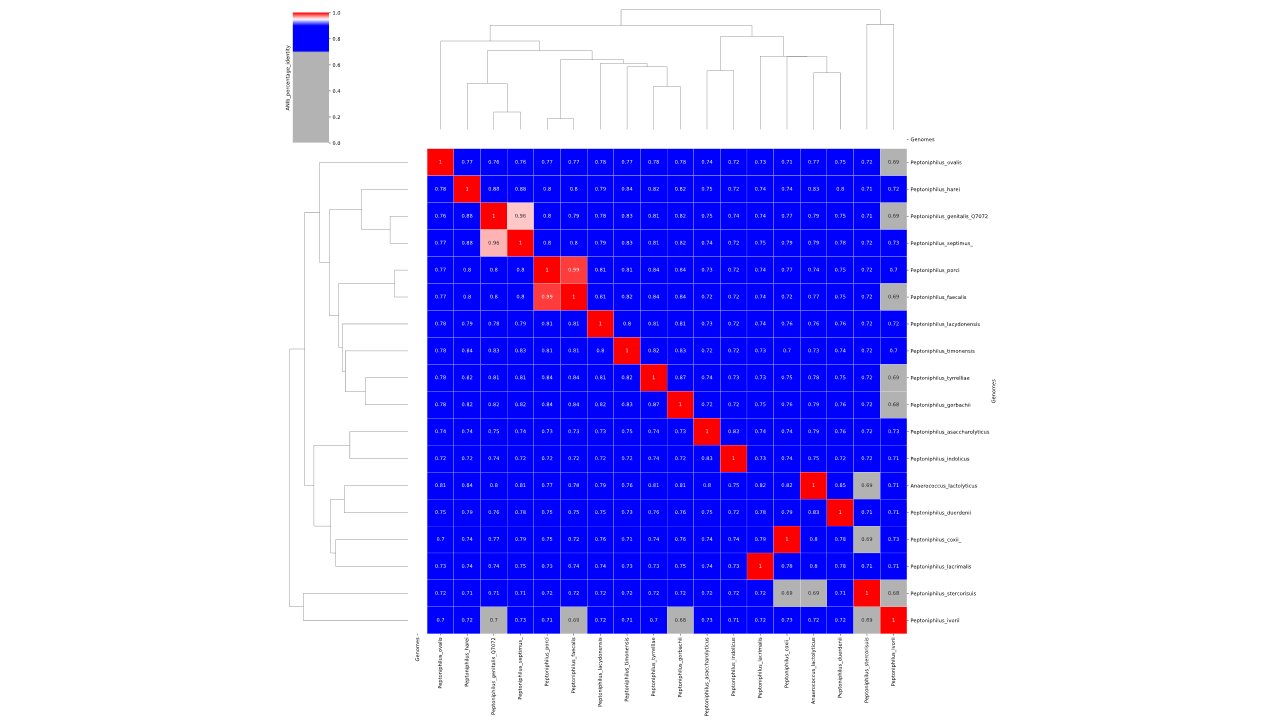

Supplement: Supplementary file 1 — Supplementary file1 (TIF 145 KB)—Heat map generated with OrthoANI values between Peptoniphilus genitalis sp. nov., strain Marseille-Q7072T and other closely related species with standing in the nomenclature. [file 284_2023_3584_MOESM1_ESM.tif]

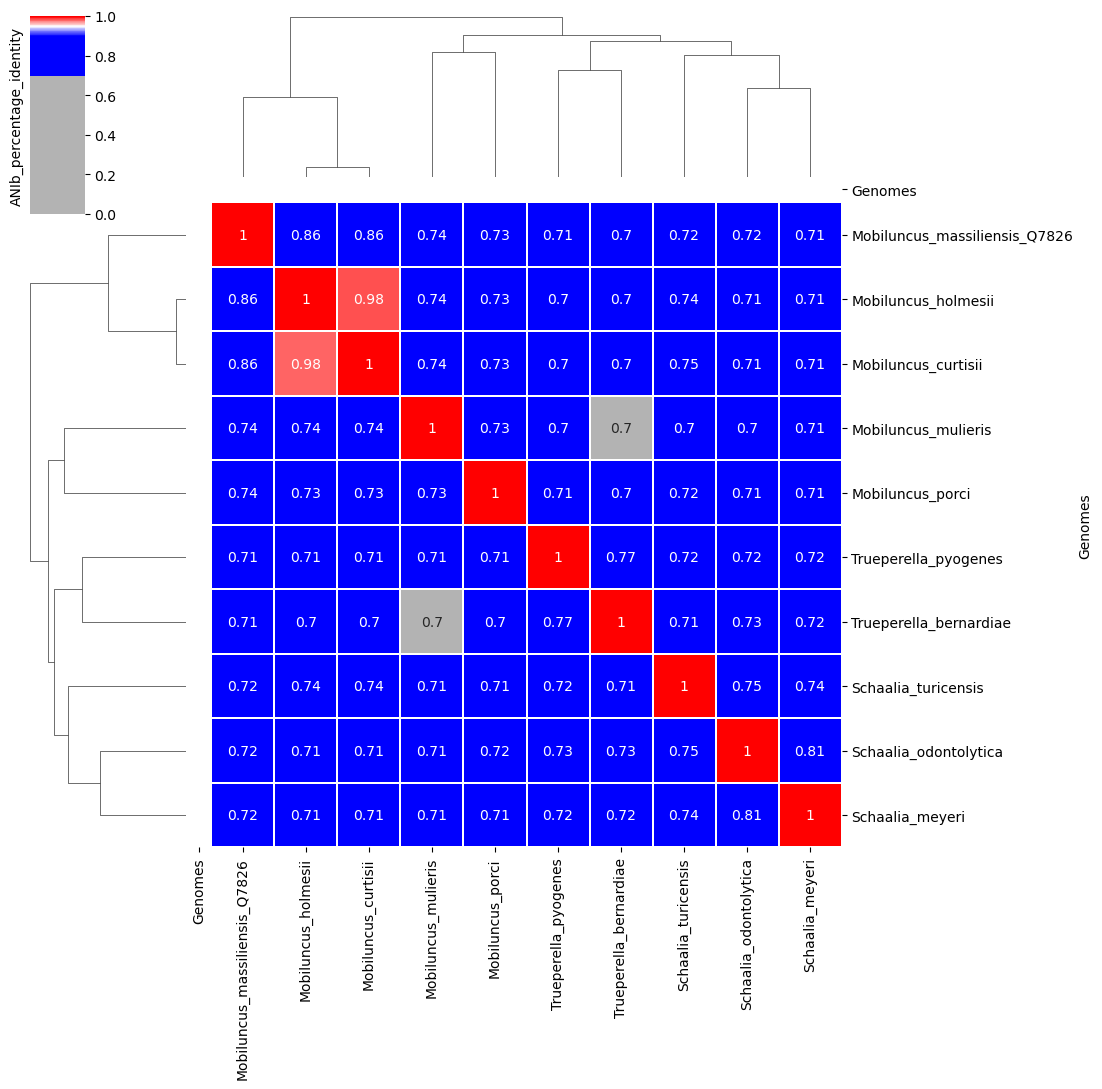

Supplement: Supplementary file 2 — Supplementary file2 (TIF 87 KB)—Heat map generated with OrthoANI values between Mobiluncus massiliensis sp. nov., strain Marseille-Q7826T and other closely related species with standing in the nomenclature. [file 284_2023_3584_MOESM2_ESM.tif]
