# Supplementary material for: Peptoniphilus genitalis sp. nov. and Mobiluncus massiliensis sp. nov.: Novel Bacteria Isolated from the Vaginal Microbiome
Source: Curr Microbiol. 2024 Feb 19;81(4):97. doi: 10.1007/s00284-023-03584-7 (PMC10876752; doi:10.1007/s00284-023-03584-7)
Supplement: Supplementary file 3 — Supplementary file3 (DOCX 21 KB) [file 284_2023_3584_MOESM3_ESM.docx]

**Supplementary Table 1.** Main criteria of strains Marseille-Q7072^T^ and Marseille-Q7826^T^.

| Properties | Strain Marseille-Q7072 | Strain Marseille-Q7826 |
| --- | --- | --- |
| Genus name | *Peptoniphilus* | *Mobiluncus* |
| Species name | *Peptoniphilus genitalis* | *Mobiluncus massiliensis* |
| Status | sp. nov. | sp. nov. |
| Designation of the type strain | CSUR Q7072 | CSUR Q7826 |
| Strain collection numbers | CECT 30604 | CECT 30727 |
| 16S rRNA gene accession number | OX458314 | OX458307 |
| Genome accession number | CATKPB000000000 | OX458329 |
| Genome size | 2,040,803 bp | 2,216,029 bp |
| G + C (mol %) | 34.3 | 57 |
| Origin | Marseille, France | Marseille, France |
| Date of isolation | 2022 | 2022 |
| Source of isolation | Human vaginal sample | Human vaginal sample |
| Conditions used for standard cultivation | Columbia agar with 5% sheep blood for 48 h of incubation | Columbia agar with 5% sheep blood for 48 h of incubation |
| Gram stain | Positive | Variable |
| Cell shape | Coccus | Curved rod |
| Cell size | 0.609 ± 0.087 μm | 1.216 ± 0.243 μm and 0.381 ± 0.039 |
| Motility | Non-motile | Motile |
| Sporulation | Non-sporulate | Non-sporulate |
| Colony morphology | Circular, gray, opaque, and convex | Circular, colorless, translucent, and convex |
| Temperature range | 20-37°C (optimum 37°C) | 20-37°C (optimum 37°C) |
| pH range | 6-8.5 (optimum pH 7) | 6-7.5 (optimum pH 7) |
| O_2_ requirement | Anaerobic and microaerophilic | Facultative anaerobic |
| Oxidase | Negative | Negative |
| Catalase | Negative | Negative |
| Salinity range | 0-5% (optimum <5%) | Without adding NaCl |

**Supplementary Table 2.** AAI values of *Peptoniphilus genitalis* sp. nov., strain Marseille-Q7072^T^ and *Mobiluncus massiliensis* sp. nov., strain Marseille-Q7826^T^ with other closely related species with standing in nomenclature.

| **Query strain** | **Subject strains** | **AAI (in %)** |
| --- | --- | --- |
| Q7072 | *“Peptoniphilus septimus”* | 95.94 |
|  | *Peptoniphilus harei* | 89.16 |
|  | *Peptoniphilus timonensis* | 85.16 |
|  | *Peptoniphilus gorbachii* | 82.80 |
|  | *Peptoniphilus tyrrelliae* | 81.69 |
|  | *Peptoniphilus porci* | 79.63 |
|  | *Peptoniphilus faecalis* | 78.65 |
|  | *Peptoniphilus lacydonensis* | 78.05 |
|  | *Peptoniphilus ovalis* | 72.07 |
|  | *Peptoniphilus lacrimalis* | 61.16 |
|  | *Peptoniphilus asaccharolyticus* | 57.56 |
|  | *Peptoniphilus duerdenii* | 57.37 |
|  | *Peptoniphilus indolicus* | 56.86 |
|  | *Peptoniphilus stercorisuis* | 56.81 |
|  | *Peptoniphilus coxii* | 52.9 |
|  | *Peptoniphilus ivorii* | 49.4 |
| Q7826 | *Mobiluncus holmesii* | 89.24 |
|  | *Mobiluncus curtisii* | 88.99 |
|  | *Mobiluncus porci* | 68.75 |
|  | *Mobiluncus mulieris* | 66.70 |
|  | *Schaalia turicensis* | 51.67 |
|  | *Schaalia odontolytica* | 50.54 |
|  | *Schaalia meyeri* | 50.01 |
|  | *Trueperella bernardiae* | 49.99 |
|  | *Trueperella pyogenes* | 49.1 |
